# Ecology and Evolution of Plants in the Mediterranean Basin: Perspectives and Challenges

**DOI:** 10.3390/plants11121584

**Published:** 2022-06-15

**Authors:** Javier Lopez-Alvarado, Emmanuele Farris

**Affiliations:** 1 Systematics and Evolution of Vascular Plants (UAB)–Associated Unit to CSIC, Unitat de Botànica, Departament de Biologia Animal, Biologia Vegetal i Ecologia, Facultat de Biociències, Universitat Autònoma de Barcelona, 08193 Bellaterra, Spain; javier.lopez.alvarado@uab.cat; 2 Department of Chemical, Physical, Mathematical and Natural Sciences, University of Sassari, Via Piandanna 4, 07100 Sassari, Italy

Preserving biodiversity at the global and local scales is a challenge for the future decades, both for protecting species and habitats and to enhance the ecosystem services they provide to the human population. There is a general agreement that biodiversity is not homogeneously distributed across the Earth surface, however, the causes promoting or affecting it and how it is differentiated among regions, ecosystems and taxa are still debated [1].

Regions with high concentration of endemic species and high levels of habitat degradation are considered biodiversity hotspots [2] at different spatial scales [3]. Five out of the twenty-five global biodiversity hotspots originally mapped [2] belong to the Mediterranean macroclimate, which is, therefore, the only macroclimate in the world having all its areas included in the biodiversity hotspots network. Among the five Mediterranean areas of the world, the Mediterranean basin is the largest one and the only characterized by having a sea in the center, including several peninsulas and thousands of islands [4,5]. This area hosts no less than 25,000 vascular plant species, of which ca. 5500 are endemic, being the third most important hotspot of the world for plant diversity.

Even if many features of the Mediterranean plant diversity are well known, including the general trajectories of evolution and diversification of plant lineages living or joining the basin in the last 5 million years [4]; several aspects of vascular plant diversity in the Mediterranean basin still need to be disentangled. Firstly, the botanical exploration is not equal in space and time for the vast and heterogeneous territories surrounding the Mediterranean Sea. Southern and eastern areas have been less explored with regard to western and northern ones, and islands with regard to continental lands. In this context, it is worth of mention the contribution by Fois et al. [6] to this Special Issue providing the first comprehensive synthesis of the vascular flora of Sardinia, the second largest Mediterranean island and the only without a recent and exhaustive list of endemic vascular plants [4,5]. As with the paper by Fois et al. [6], our Special Issue gives an important contribution to the improvement of knowledge of the role that Mediterranean island systems play for the differentiation of endemic lineages of vascular plants, laying the foundation for their future conservation.

At the local scale, vascular plant species richness and assemblages’ diversity are influenced by meso-climate features of the different territories and their geomorphology; in particular elevation gradients and mountain distribution have a crucial role. However, there are not many papers dealing with the effects of elevation gradients on vascular plant diversity; Di Biase et al. [7] sheds light on this intriguing aspect of Mediterranean plant diversity, whose importance was already underlined by Thompson [4]. Di Biase et al. [7] describe the variation of the ecological (life-forms) and the geographical (chorologic types) contingents of the Mt. Genzana (central Apennines, Italy) flora, showing the increase of the endemic component with the increase of the elevation. This research emphasizes the need of local studies for knowing and conserving plant diversity in the Mediterranean area, and the pivotal role of mountain chains and massifs where the Mediterranean bioclimate interacts with the Euro-Siberian one.

This is true also for species living along altitudinal gradients where the Mediterranean makes contact with Euro–Siberian or Alpine areas, as explored by Fontaine et al. [8] studying the niche variation of *Lilium pomponium* L. (Liliaceae) on a wide altitudinal gradient in the Maritime Alps, as a paradigmatic situation common to a wide variety of Mediterranean vascular plants that, despite their narrow ranges, exhibit high levels of ecological diversity in terms of occupied niches [9]. The paper by Fontaine et al. [8] highlights that knowledge of the fine-scaled ecological conditions that determine niche types are essential for conservation management of the habitats of Mediterranean endemic species and for the exploration of their possible response to ongoing climate change.

Another aspect still to be disentangled is the role of small and micro island systems in conditioning the population structure and the reproductive performance of Mediterranean vascular plants. The basin hosts, in fact, hundreds of islands and thousands of small or very small islands, whose role in the differentiation of plant lineages is poorly known. In the Special Issue, Murru et al. [10] investigates this aspect taking *Silene velutina* Loisel. (Caryophyllaceae) as a model species. This plant is endemic of a few sites in Sardinia and Corsica, where it grows on both the two large islands and several islets. The results presented by Murru et al. [10] showed that the ecological context, the population structure, and the reproductive performance are significantly different among populations living in small and large islands, conditions that determine niche types are essential for conservation management of the habitats of Mediterranean endemic species and their possible response to ongoing climate change.

However, many aspects of plant population dynamics and its role in enhancing the evolutionary potential of novel lineages remain to be fully understood; for example, the contribution to genetic diversity from single populations of narrowly distributed plant taxa [11]. As a model species for this important aspect of Mediterranean plant diversity, the north African (Algeria and Tunisia) *Medicago tunetana* (Murb.) A. W. Hill (Fabaceae) showed a large genetic diversity among populations despite its narrow distribution range [12]. This pattern of intra-specific high genetic diversity among populations of narrow endemic Mediterranean plants is a common feature in many genera and families [13] and should be taken into account when planning conservation and management strategies.

Under the ecological point of view, the Mediterranean can be considered a complex, multi-hierarchical system of islands-within-islands, where beyond geographical islands (small land patches surrounded by the sea), there are several other types of islands (climatic islands, edaphic islands, ecosystem islands). This pronounced patchiness has a strong influence on the presence, extent, density, and structure of plant populations, strongly conditioning their conservation. This aspect is deepened by Cogoni et al. [14] on the pterydophyte *Ophioglossum vulgatum* L. (Ophioglossaceae), whose distribution is characterized by the presence of extremely small and fragmented populations, conditioned by the different water availability in space and time.

Finally, last but not least, an increasing attention should be paid by future investigations to the contribution given by interactions between vascular plants and other organisms, especially those able to establish bio-chemical interactions as viruses, bacteria, and fungi. This aspect is explored in our Special Issue by Muresu et al. [15], who investigated the interactions among some Fabaceae species and symbiotic and endophytic bacteria in Sardinia. This paper shows an intriguing field of plant biodiversity research, with important applications to understand the trajectories of plant evolution as strongly conditioned by interactions among plants and other organisms.

Overall, our Special Issue opens a window not only on the state-of-the-art of scientific research on plant diversity in the Mediterranean, but also on crucial and promising future developments. There is increasing evidence that biological diversity is not a static concept but needs to be continuously monitored at multiple spatial scales, from regional [6], to local [7], to detailed niche adaptation [10]. If we go further to the very detailed, even organisms, such as plants and animals, can be considered at spatial scale as we are aware that they (we) are true ecosystems [15]. The comprehension of biodiversity at multiple spatial scales is, therefore, a crucial challenge for the research on plant ecology and the evolution of Mediterranean plants in future decades. The same is true regarding both ecological [8] and genetic diversity among populations of the same species [12]. In an ecologically and geographically fragmented system, such as the Mediterranean basin, this will be increasingly jeopardized by global climatic change; the role of single populations as particular objects of plant adaptation and evolution will be crucial, not only for their conservation [14], but also for the ecosystem services they provide to humanity.
